# Localised erythema nodosum leprosum—A rare entity managed with thalidomide

**DOI:** 10.1002/ski2.339

**Published:** 2024-02-07

**Authors:** Chandni Patel, Barbara De Barros, Stephen L. Walker

**Affiliations:** ^1^ Department of Healthcare Services for Elderly People Royal Free Hospital NHS Foundation Trust London UK; ^2^ Hospital for Tropical Diseases University College London Hospitals NHS Foundation Trust London UK; ^3^ Faculty of Infectious and Tropical Diseases London School of Hygiene and Tropical Medicine London UK; ^4^ Department of Dermatology University College London Hospitals NHS Foundation Trust London UK

## Abstract

Leprosy is caused by *Mycobacterium leprae*. The condition primarily affects the skin and peripheral nerves. There are two types of leprosy reactions, Type 1 and Type 2 or erythema nodosum leprosum (ENL). ENL is a severe multi‐system, immune‐mediated complication of lepromatous leprosy. It is characterised by widespread painful cutaneous nodules, fever and peripheral oedema. This report discusses the unusual case of a 29‐year‐old woman who developed a localised form of ENL which required thalidomide to induce remission.

## Graphical abstract

Erythema nodosum leprosum is a severe multi‐system, immune‐mediated complication of lepromatous leprosy. It is characterised by widespread painful cutaneous nodules, fever and peripheral oedema. This report discusses the unusual case of a 29‐year‐old woman who developed a localised form of ENL which required thalidomide to induce remission.

## 1 CASE REPORT

A 29‐year‐old woman was diagnosed with lepromatous leprosy (LL) and Type 1 reaction (T1R). Full blood count, renal function, liver function, thyroid function and HbA1c were normal. ANA, ANCA, HIV, Hepatitis B and C, treponemal and Strongyloides serologies were negative. The skin biopsy showed sheets of macrophages in the dermis located around and within nerves and skin adnexal structures without discrete granulomas. The bacterial index (BI) was six with globi present. The BI is a semi‐logarithmic scale of the density of *M. leprae* bacilli per oil immersion field; in LL bacilli may be grouped in clusters known as globi.^1^

The patient was treated with 12 doses of monthly rifampicin, ofloxacin and minocycline and required oral prednisolone for 18 months to manage the T1R. She experienced intermittent swelling of the legs, particularly the right following completion of anti‐microbial treatment. The skin of the distal right leg was sclerotic, hyperpigmented and anaesthetic.

**FIGURE 1:**
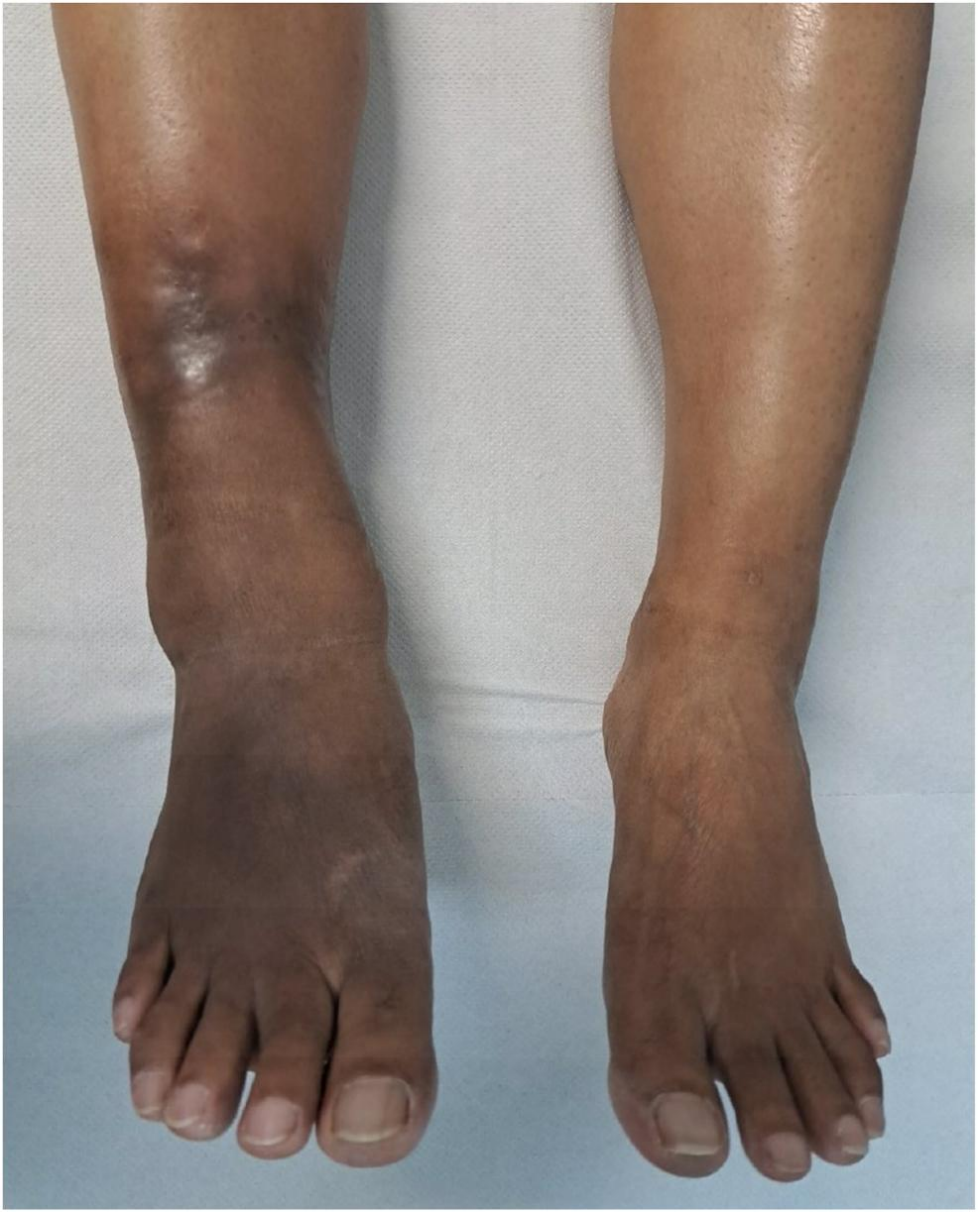
Hyperpigmented plaque on right leg with nodules of ENL.

Thirteen months after stopping prednisolone she developed oedema and very painful, red nodules on the right leg, medial and plantar surfaces of the right foot in the anaesthetic hyperpigmented plaque which had previously been affected by T1R (Figure 1). Walking was very uncomfortable. She was afebrile and no other body sites were affected. A diagnosis of ENL was made based on the morphology of the skin lesions and the marked tenderness. A skin biopsy was not performed as a confident clinical diagnosis was made and the location of the lesions and sclerotic nature of the skin of the distal leg posed a significant unwarranted risk of prolonged delayed healing.

The ENLIST ENL Severity Scale (EESS) score was six.^2^ Slit‐skin smear showed the mean BI was two.

**FIGURE 2:**
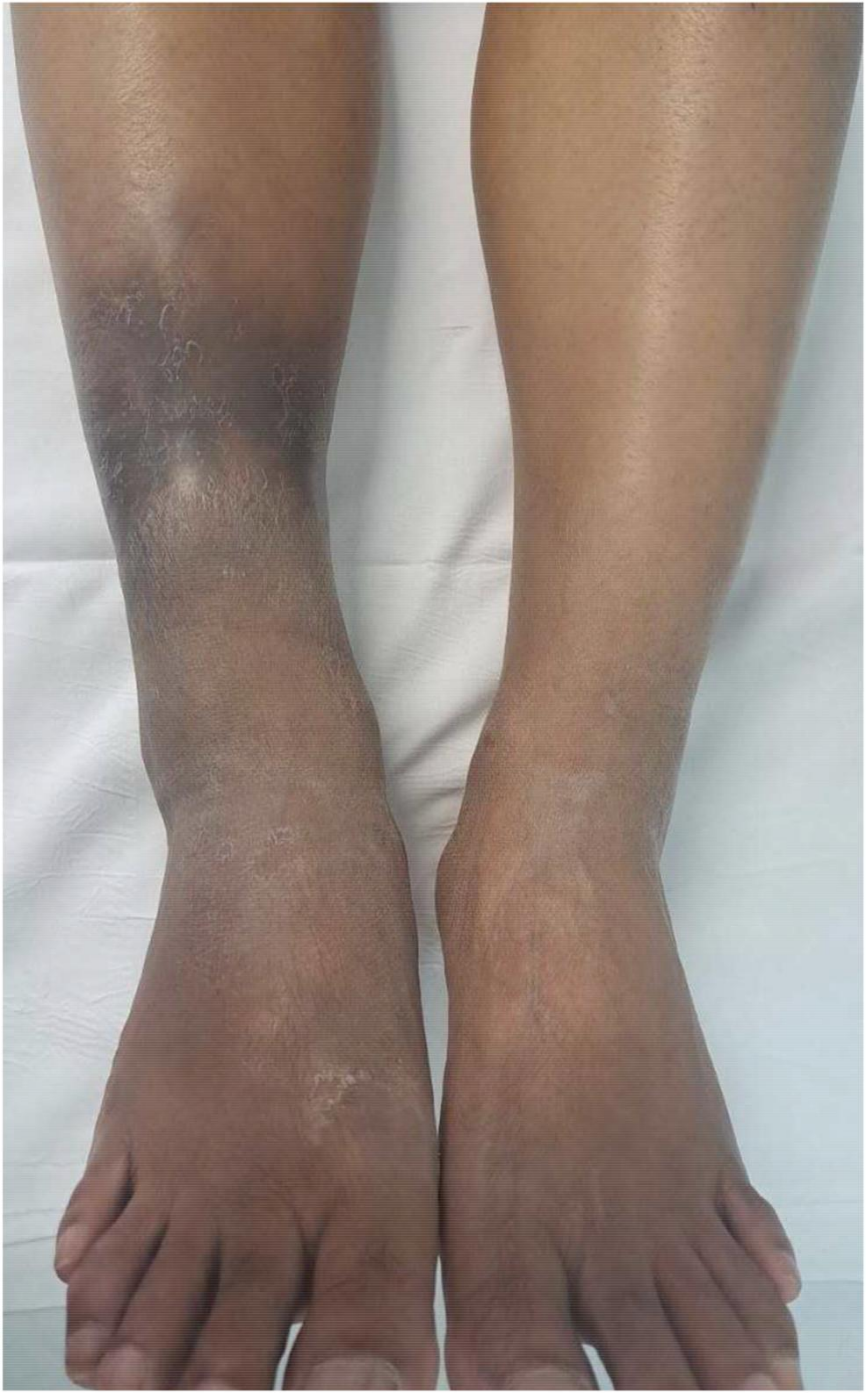
Right leg with no oedema or painful nodules while taking thalidomide 150 mg daily.

The patient was reluctant to resume prednisolone. Oral ibuprofen was prescribed with no effect and so prednisolone 20 mg daily was commenced with rapid improvement in pain and resolution of the nodules. Reducing the dose of prednisolone resulted in recurrence of the painful nodules at the same site. She had an episode of swelling of her fingers. Due to the lengthy exposure to corticosteroids and following detailed counselling, appropriate consent and starting an oral contraceptive (in addition to using a barrier contraceptive method) the patient started thalidomide 200 mg daily. The prednisolone was gradually reduced to zero. There was rapid and complete control of her symptoms until she inadvertently ran out of thalidomide. The nodules recurred and the EESS score was eight. This ENL flare resolved rapidly on resuming thalidomide (Figure 2).

There was no deterioration of the localised ENL on slowly reducing the dose of thalidomide. However, unsurprisingly there was no improvement in the longstanding thickening, hyperpigmentation or anaesthesia of the skin of the right leg.

Leprosy is caused by *Mycobacterium leprae*. The condition primarily affects the skin and peripheral nerves. *M. leprae* infection is readily treatable with multi‐drug therapy but 30%–50% of patients with leprosy can develop immune‐mediated reactions affecting the skin, peripheral nerves and other organs.^3^ Reactions are a major risk factor for leprosy associated disability.

ENL is a severe multi‐system, immune‐mediated complication of LL.^4^ It is characterised by widespread painful crops of new cutaneous and subcutaneous nodules, fever and peripheral oedema.^5^ Arthritis, iritis, orchitis and neuritis may occur. LL and a mean BI greater than or equal to four are risk factors for the development of ENL.^6^ Approximately 50% of individuals with LL develop ENL. Thalidomide is highly effective and has been demonstrated to induce a faster clinical response (cutaneous and systemic) and reduce the number of relapses compared to prednisolone.^7^ T1Rs in contrast are typified by oedematous, erythematous plaques occurring in pre‐existing leprosy skin lesions, peripheral oedema and neuritis may occur but no other organs are affected.^8^

Darlong et al. described the use of thalidomide in those with ENL living in rural India. The report recommended utilising thalidomide in preference to prednisolone in chronic ENL to avoid corticosteroid adverse effects such as diabetes mellitus, hypertension, infections and the risk of chronic adrenal suppression.^9^

Thalidomide is not effective in the management of T1R.^10^ Thalidomide is associated with adverse effects including teratogenicity.^7^, ^11^

The successful and safe use of thalidomide in women with ENL between 1998 and 2014 was previously reported from our centre. Thirty individuals with ENL, 10 of whom were women, were treated with thalidomide after a median period of eight months. The median duration of ENL for both men and women in the cohort was 60 months (Range 6–192 months).^12^

There have been reports of less common variants of ENL skin lesions such as a bullous, pustular, necrotic and ulcerated forms^5^ and ENL involving the orbit resulting in orbital ischaemia.^13^ Reports of localised ENL are rare but areas of ENL associated chronic panniculitis may lead to fixation of the skin and subcutaneous tissue to deeper structures.^14^ Inflammation of the subcutaneous fat may explain the pain experienced by our patient despite the loss of light touch, pin prick and temperature sensation, due to damaged dermal nerve fibres, within the hyperpigmented skin of the right leg.

A case report by Prabhu et al. described a presentation of ENL as localised but the erythematous, painful papules and nodules on the posterolateral aspects of both thighs were associated new nodules on the face and upper extremities. The presentation described appeared to be in keeping with the widespread cutaneous lesions commonly seen in ENL.^15^

Localised ENL may be difficult to diagnose due to the rarity and limited extent of disease in contrast to the typical generalised nature of ENL. The localised form we report here appears to follow a chronic course as is commonly seen in ENL and despite being localised required treatment with thalidomide.

## CONFLICT OF INTEREST STATEMENT

The authors have no conflicts to declare.

## AUTHOR CONTRIBUTIONS

**Chandni Patel**: Writing – original draft (lead); writing – review & editing (lead). **Barbara De Barros**: Supervision (supporting); writing – review & editing (supporting). **Stephen L. Walker**: Supervision (lead); writing – review & editing (supporting).

## FUNDING INFORMATION

Payment of publication fees was supported by the Hospital for Tropical Diseases Charitable Fund which had no role in decision to publish, or preparation of the manuscript.

## ETHICS STATEMENT

No ethical approval was required.

## Data Availability

Data sharing is not applicable to this article as no new data were created or analysed in this study.
